# Differential associations of the two higher-order factors of mindfulness with trait empathy and the mediating role of emotional awareness

**DOI:** 10.1038/s41598-023-30323-6

**Published:** 2023-02-24

**Authors:** Olaf Borghi, Lukas Mayrhofer, Martin Voracek, Ulrich S. Tran

**Affiliations:** Department of Cognition, Emotion, and Methods in Psychology, Faculty of Psychology, University of Vienna, Liebiggasse 5, 1010 Vienna, Austria

**Keywords:** Psychology, Human behaviour

## Abstract

Empathy enables us to understand the emotions of others and is an important determinant of prosocial behavior. Investigating the relationship between mindfulness and empathy could therefore provide important insights into factors that promote interpersonal understanding and pathways that contribute to prosocial behavior. As prior studies have yielded only inconsistent results, this study extended previous findings and investigated for the first time the associations of two important factors of mindfulness (Self-regulated Attention [SRA] and Orientation to Experience [OTE]) with two commonly proposed components of empathy (cognitive empathy and affective empathy). Using a community sample of *N* = 552 German-speaking adults, the two mindfulness factors were differentially associated with cognitive and affective empathy. SRA correlated positively with cognitive empathy (*r* = 0.44; OTE: *r* = 0.09), but OTE correlated negatively with affective empathy (*r* = − 0.27; SRA: *r* = 0.11). This negative association was strongest for one specific aspect of affective empathy, emotional contagion. Revisiting previously reported mediating effects of emotion regulation, we found that emotional awareness mediated the associations with both components of empathy, but only for SRA. Together, these findings imply that mindfulness benefits the cognitive understanding of others’ emotions via two distinct pathways: by promoting emotional awareness (SRA) and by limiting the undue impact of others’ emotions on oneself (OTE).

## Introduction

Mindfulness is a concept with roots in ancient Buddhist meditation traditions^1,2^ and in the last decades has become a major topic in psychology^3^, with numerous studies reporting beneficial outcomes of mindfulness on clinical^4^, cognitive^5^, and prosocial processes^6^. A common definition describes mindfulness as intentionally maintaining attention on the present moment while adopting a nonjudgmental stance^1^. In a functional analytic way, the form of contact with the present moment in mindfulness can further be described as defused, accepting, and open, with a person noticing the experiences in the present moment, but with a transcendent sense of self^7^. Bishop and colleagues emphasized the dimensionality of the construct and proposed two components^3^: Self-regulated Attention (SRA; the ability to bring one’s attention to the present moment and to intentionally switch attention between objects) and Orientation to Experience (OTE; an open, accepting, curious, and nonjudgmental attitude towards own experiences and the world). This definition may prove particularly useful, as the two components likely also relate to two important meditation styles^8^. Focused Attention meditation involves selectively maintaining and effortfully bringing back one’s moment-to-moment attention to a selected object, and this style appears to be associated with the cultivation of SRA^8,9^. Another meditation style, Open Monitoring meditation, involves non-reactive, effortless monitoring of one’s moment-to-moment experience, without explicitly selecting and maintaining focus on a particular object. This style appears to be associated with the cultivation of OTE^8,9^. This link between the two components of mindfulness and specific meditation styles could allow tailor-made interventions (training with a focus on one meditation style) that could have the potential to not only promote the associated mindfulness-component, but possibly also other related outcomes.

Empathy can be defined as a skill or trait that enables individuals to vicariously experience and understand the emotions of others^10,11^. There are many different definitions and conceptualizations regarding the structure of empathy^12^. One common definition suggests two interrelated components: Affective empathy describes the ability to vicariously experience others’ emotions, whereas cognitive empathy describes the processes that enable one to understand and interpret one’s own and others’ emotions^10,11^. Other definitions proposed emotion regulation^13^ or prosocial motivation^14^ as a third additional component, two concepts that are considered as strongly related to, but that are deliberately separated from empathy in the two-component model^11^. In general, empathy is viewed as a determinant of prosocial behavior and may enable us to respond appropriately to the needs of others or may motivate us to help, which could benefit our social relationships^14,15^.

Associations between mindfulness and empathy could provide important insights into factors underlying or promoting prosocial behavior and thus warrant further investigation. However, research on both mindfulness and empathy is surrounded by methodological and conceptual issues, which may give rise to inconsistent results^12,16^. This is also reflected in the extant literature on their mutual relationship.

Prior studies reported positive associations of mindfulness with components of cognitive empathy^17–20^, but the association of mindfulness with components of affective empathy varied widely, from positive^17,21^, null^18^, to negative^19,20^. A recent meta-analysis of studies on the relationship between mindfulness and empathy (as measured with the Interpersonal Reactivity Index^22^; IRI) in samples of counseling professionals reported an overall positive association of mindfulness with perspective taking, and a negative association with personal distress^23^. However, in many of these studies, either mindfulness, empathy, or both constructs were measured with unidimensional scales, even though definitions and empirical findings indicate multidimensionality^9,11^. More recent studies investigated the associations of mindfulness and empathy also on their subscale levels. Yet, associations still await their contextualization into the two-component models of mindfulness and empathy.

MacDonald and Price^24^ reported medium-to-high correlations between the subscales of the Five Facet Mindfulness Questionnaire (FFMQ)^25^ with cognitive and affective empathy, as measured with the Questionnaire of Cognitive and Affective Empathy (QCAE)^11^. The FFMQ measures mindfulness with five facets (i.e., subscales): Observe (actively perceiving internal and external stimuli), Describe (the ability and tendency to describe internal experiences with words), Actaware (being attentive towards one’s own actions), Nonjudge (taking a neutral, non-judgmental stance towards one’ own thoughts and feelings), and Nonreact (attending to feelings and thoughts without being carried away by them). Observe, Describe, and Nonreact correlated positively with cognitive empathy, and Describe, Nonreact, Actaware, and Nonjudge correlated negatively with affective empathy. These correlations were partially mediated by alexithymia (i.e., difficulties in recognizing, labeling, and processing emotions), highlighting the importance of emotional awareness for empathic processes.

Fuochi and Voci^26^ investigated associations between the subscales of the FFMQ and the IRI. All five facets of mindfulness correlated positively with perspective taking. Observe, Describe, and Actaware correlated positively with the subscale emotional concern, a result that is partially in line with a previous study that reported a positive association between the facets Observe and Describe and emotional concern^27^, and all facets except Observe correlated negatively with personal distress. Further, they expanded the extant findings on specific mediational paths. They reported an indirect effect of mindfulness on empathy via emotion regulation processes as measured with the Difficulties in Emotion Regulation Scale (DERS)^28^. While emotional awareness (the tendency to be aware of, acknowledge, and attend to emotions) mediated the associations of Observe, Describe, and Nonreact with the components of empathy, impulse control (the tendency to remain in control of one’s behavior when experiencing negative emotions) and emotion regulation strategies (the belief of being able to effectively regulate emotions when one is upset) mediated the respective associations with Actaware, Nonjudge, and Nonreact. Such associations between mindfulness, empathy and emotion regulation abilities are also in line with studies reporting a positive relationship between self-reported mindfulness and trait emotional intelligence (TEI)^29,30^, i.e., emotion-related behavioral dispositions and self-perceptions, including but also going beyond concepts such as trait-empathy and self-perceived emotion regulation abilities^31^.

Still, even these studies are subject to some further methodological limitations. First, extant research considered no or only a limited number of potential background confounders. Meditation experience was reported to moderate the associations of mindfulness with other constructs, including empathy^26,32^. Additionally, there also are sex differences in empathy^33,34^. However, when investigating indirect effects, only MacDonald and Price^24^ controlled for sex differences and neither study considered participants’ meditation experience.

Adding to this, across many studies^17–19,21,24,26^, only limited emphasis was put on delineating empathy from related concepts. This remains an important research desiderate, especially regarding emotional contagion. In empathy, the source of the emotion is recognized to lie outside rather than within the self (referred to as self/other distinction)^15^. While emotional contagion surely contributes to the empathic process, a high tendency to “catch” the emotions of others could be grounded in a limited ability to differentiate between the self and others, thus making it partly different from empathy^15^. Accordingly, high levels of emotional contagion have even been associated with negative outcomes, such as impaired emotion regulation, neuroticism, abnormal eating behavior and general psychological vulnerability^33,35,36^. High emotional contagion appears to be especially harmful in emotionally negative contexts, as individuals could become overwhelmed by the emotions of others, resulting in emotional distress^37^. Psychological benefits of empathy might thus specifically depend on the absence of high levels of emotional contagion. Negative associations between self-reported mindfulness and emotional distress, a potential outcome of high emotional contagion, were reported as well^23,30^, and a lower susceptibility to emotional contagion was proposed as a potential pathway between mindfulness, less anxiety symptoms, and a lower risk for burnout in health care professionals^38,39^. Therefore, one could expect negative associations of mindfulness specifically with emotional contagion, which could also have some relevance as a link between mindfulness and beneficial mental health outcomes^4,38^.

Other, more general limitations were mainly related to the applied self-report measures. Most studies either assessed mindfulness using the Mindfulness Awareness Attention Scale (MAAS)^40^ or the FFMQ. While both measures demonstrate good psychometric properties^40,41^, the MAAS has been criticized for being partially inconsistent with mindfulness definitions. Its items capture a unidimensional construct of mind*less*ness (emphasis ours): It therefore appears to reflect more strongly a (negative) element of mindfulness related to absent-mindedness, compared to other measures that may capture a broader subset of (positive) aspects of mindfulness^25,42^. The FFMQ on the other hand provides a more comprehensive picture of the construct. The five-facetted structure derives from empirical findings^25,41^, which also poses some challenges in linking it to (other) theoretical assumptions about, or definitions of, mindfulness (e.g., the two-component model^3^). Advantageously, the facets of the FFMQ have also been shown to fit well with a two-factor higher-order structure associated with SRA and OTE^9^, combining its empirical strengths with the advantages of the two-component model^3^. The factorial validity of these two higher-order factors was recently replicated, and differential associations of the two factors with mental health outcomes in meditating and nonmeditating samples were suggested^32,43^. Extracting two higher-order factors from the FFMQ facets could thus offer an efficient, empirically, as well as theoretically supported operationalization of mindfulness. This approach remains yet to be applied when investigating associations with empathy and may provide information on the specific pathways along which mindfulness is associated with it.

The most commonly applied self-report measure to assess empathy was the IRI. However, the subscales of the IRI were criticized to possibly confound empathy with other constructs, such as imagination and emotional self-control^11,37,44^. In contrast, the QCAE is a well-established self-report instrument of empathy, but has so far only rarely been used in the context of mindfulness research^20,24^. The QCAE delineates empathy better than the IRI from reactive emotions, such as sympathy, and its structure is in line with up-to-date definitions of two higher-order factors, each of which is measured with multiple subscales. This allows for both precise and general statements about the associations of other constructs with the components of empathy^11,33,45^, specifically emotional contagion. However, the associations of the subscales of affective empathy (i.e., Emotion Contagion, Proximal Responsivity, and Peripheral Responsivity; see “Methods”) with mindfulness have not yet been investigated.

In summary, recent large-scale studies have suggested several links and pathways between mindfulness and empathy^24,26^. Still, the relationship between mindfulness and empathy is far from being clear and due to methodological and conceptual limitations their relationship requires further research. To provide insight both on a theoretical and empirical level, investigations should be guided by theoretical conceptualizations of the two constructs, such as the two-component model of mindfulness, but also probe associations between sub-components that could explain heterogeneity in previous results, such as with emotional contagion. Other methodological aspects, such as considering different background confounders, need further attention as well. In addition, reported mediating effects of emotion regulation abilities between mindfulness and empathy could be fruitfully re-investigated in a study that aims to account for these previous limitations. Together, this could provide much needed clarity on the interrelation of mindfulness and empathy and pave the way forward for future research or interventional approaches. This motivated the present research.

### The present research

Our first research goal aimed at replicating the higher-order two-factor structure for the FFMQ^9,32,43^, in order to operationalize mindfulness in line with the two-component model^3^. Following a replication-extension approach^46^, the second research goal aimed at extending previous findings by investigating the associations of these two higher-order factors of mindfulness with components of empathy in detail.

**Figure 1:**
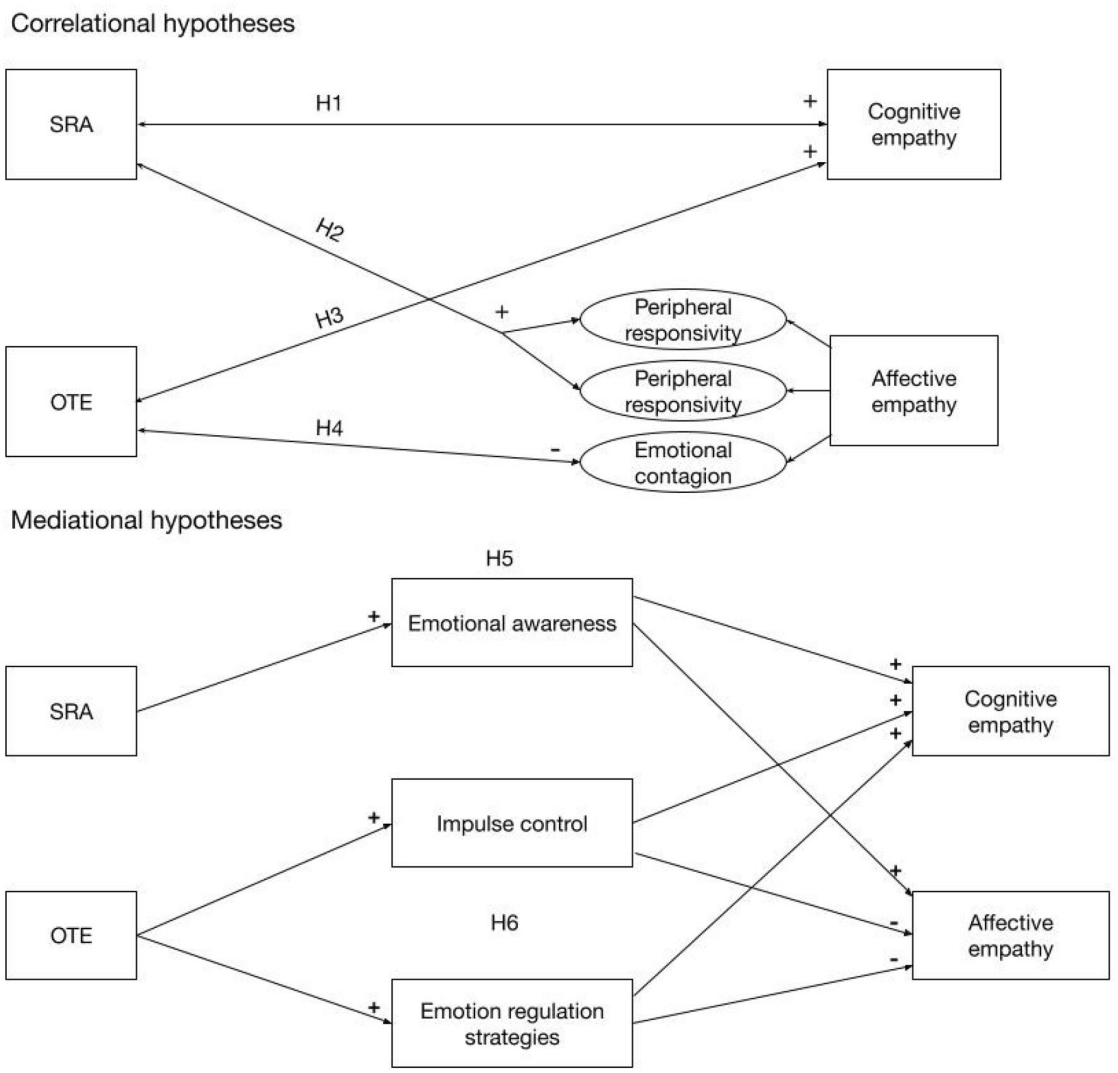
Overview of the a-priori hypotheses. SRA = Self-regulated Attention, OTE = Orientation to Experience. Meditation experience and participants’ sex were controlled as background confounders.

We thus had several hypotheses for the present study (for a summary visualization set of hypotheses, see Fig. 1): We expected that SRA will correlate positively with cognitive empathy (Hypothesis 1) and affective empathy (i.e., QCAE subscales Peripheral and Proximal Responsivity), but not with emotional contagion (QCAE subscale Emotion Contagion; Hypothesis 2). We further expected that OTE will correlate positively with cognitive empathy (Hypothesis 3), but negatively with affective empathy, especially with its sub-component emotional contagion (Hypothesis 4).

As the third research goal, we aimed to conceptually replicate mediational effects of emotion regulation abilities between mindfulness and empathy, as previously reported by Fuochi and Vochi^26^. We expected that the relationships between SRA and cognitive and affective empathy will be mediated by the DERS subscale Emotional Awareness (Hypothesis 5) and that the relationships between OTE and cognitive and affective empathy will be mediated by the DERS subscales Impulse Control and Emotion Regulation Strategies (Hypothesis 6).

Further, we controlled for the possible confounders participant sex and meditation experience. In an exploratory analysis, we additionally investigated the effects segmented by sex, and in a supplemental analysis, for better comparability with previous studies, we investigated the mediational effects also on the facet level of mindfulness.

## Methods

### Participants

The sample for this study consisted of a total of *N* = 552 German-speaking participants from the general adult population (67% women; age: *M* = 31.8, *SD* = 14.2, range: 18–78 yrs.). 34% of participants reported meditating at least once a week and thus could be considered regular meditators^41^. Further sample characteristics are provided in Table 1.

**Table 1:** Sample characteristics.

Characteristic	Full sample	
	*n*	%
Sex		
Female	369	66.8
Male	178	32.2
Other/not specified	5	0.9
Nationality		
Austria	257	46.6
Germany	198	35.9
Other European country	97	17.6
Highest educational level		
Compulsory/vocational education	82	14.9
Upper secondary education	259	46.9
Tertiary education	211	38.2
Currently studying	286	51.8
Currently employed	357	64.7
Meditation experience		
At least once a week (regular meditators)	186	33.7
At least once a month	53	9.6
Less frequent or no experience at all	313	56.7
Most common types of meditation practice^a^		
Yoga	121	50.6
Zen	14	5.9
Transcendental meditation	14	5.9
Other	90	37.6

*N* = 552 German speaking adults.

^a^Among all participants meditating at least once a month (*n* = 239).

### Procedure

Participants were recruited by several study inviters via social media and personal contacts to partake in an online survey created with SoSci Survey^47^ that consisted of 13 scales in total (as assembled for a larger research project), five of which were relevant for the present study. An overview over all scales administered, as well as their respective position within the survey, is provided online (see “Open practices” section, below).

Data collection took place during April 2021. Participants had to be at least 18 years old, proficient in the German language, and provided informed consent before inclusion in the study. Participation was anonymous and voluntary. As an incentive, participants could opt-in to partake in a raffle with the possibility of winning one of two 25€ coupons. Contact information (email addresses) was electronically stored separately from all other participant data to guarantee anonymity and irrecoverably deleted after the raffle closed.

In total, *N* = 850 persons accessed the online survey, of which *n* = 558 filled out all five scales that were relevant for this study. Three cases had to be excluded as they stated being younger than the required age limit; two more cases were excluded for more than 5% missing items in at least one of the relevant scales; one case had to be excluded as, judging from the time count provided by the online survey, the participant had merely rushed through the survey, indiscriminately selecting the same response alternative for most items. Six individual missing values in the remaining data were replaced by the respective individual scale means.

We utilized the safeguard power-analytic approach to perform a conservative (i.e., realistic, if not “calculated pessimistic”) a priori power analysis^48^, using G*Power^49^. Following this approach, we did not select empirically observed effect sizes as the target effect size for this study, but rather (in the manner of reasonable calculated pessimism) the lower bound of the 60% confidence interval (*r* = 0.13) of the most conservative estimate of the association between mindfulness and empathy available from prior related research^24,26^ (namely, *r* = 0.16). This power analysis (two-tailed α = 0.05; desired power 1 – β = 0.80) suggested a sample size of at least *N* = 462 necessary for an effect of this size. A sample size of *N* = 462 was also found to achieve a power of 0.80 in a mediation analysis, using a bias-corrected bootstrapping procedure for relevant small-to-medium effects (i.e., standardized estimates = 0.14)^50^. This study’s sample size thus was deemed appropriate to detect target effects of relevant size.

### Measures

All scales were presented in German. Sample reliabilities internal consistency were calculated according to McDonald’s ω and Cronbach’s α, using the MBESS package in R^51^, and are reported in Table S1 in the Supplementary Materials.

#### Five Facet Mindfulness Questionnaire

Mindfulness was measured with a 23-item short form^32^ of the 39-item Five Facet Mindfulness Questionnaire^25^. Items were rated on a 5-point Likert scale ranging from 1 = *never or very rarely true* to 5 = *very often or always true*. Four items each measured the facets Observe (e.g., “I pay attention to sensations, such as the wind in my hair or sun on my face”), Describe (e.g., “I’m good at finding words to describe my feelings”), Actaware (e.g., “I am easily distracted”; reverse-scored), and Nonjudge (e.g., “I tell myself I shouldn’t be feeling the way I’m feeling”; reverse-scored); Nonreact was measured with all seven items of the full form (e.g., “In difficult situations, I can pause without immediately reacting”). The FFMQ demonstrated high construct validity in different samples^41^, and the German short form showed improved psychometric properties relative to the full form^9^.

#### Questionnaire of Cognitive and Affective Empathy

Empathy was measured with the Questionnaire of Cognitive and Affective Empathy^11^. The QCAE has a total of 31 items, which are rated on a 4-point Likert scale ranging from 1 = *strongly disagree* to 4 = *strongly agree*. It assesses the two dimensions of cognitive and affective empathy on five subscales: Perspective Taking (the ability to intuitively put oneself in situations of others and interpret these from their perspective; with items such as “I can pick up quickly if someone says one thing but means another”), Online Simulation (attempting to put oneself in another person’s position to understand their feelings and anticipate future actions; with items such as “I find it easy to put myself in somebody else’s shoes”), Emotion Contagion (automatically mirroring other people’s emotions; with items such as “People I am with have a strong influence on my mood”), Proximal Responsivity (the affective responsivity when other people’s feelings are observed in a close social context; with items such as “I often get emotionally involved with my friends’ problems”), and Peripheral Responsivity (the affective responsivity when other people’s feelings are observed in a more detached context, like observing characters in a movie; with items such as “I usually stay emotionally detached when watching a film”, reverse-scored). The first two subscales measure cognitive empathy, and the latter three subscales measure affective empathy. The QCAE demonstrated good psychometric properties with good convergent and construct validity^11^. High validity and reliability were also replicated in the German translation of the QCAE^45^.

#### Difficulties in Emotion Regulation Scale

Aspects of emotion regulation were measured using three of the six subscales of the Difficulties in Emotion Regulation Scale^28^: Lack of Emotional Awareness (six items; e.g., “I pay attention to how I feel”, reverse-scored), Limited Access to Emotion Regulation Strategies (eight items; e.g., “When I’m upset, it takes me a long time to feel better”), and Impulse Control Difficulties (six items; e.g., “When I’m upset, I become out of control”). Items were rated on a 5-point Likert scale ranging from 1 = *almost never* to 5 = *almost always*. The DERS has shown to be valid across non-clinical and clinical populations also in the German translation^52^. High DERS scores reflect difficulties in emotion regulation. In this study, DERS subscales were reverse-scored to allow for an easier interpretation and to thereby reflect emotion regulation abilities (i.e., emotional awareness, emotion regulation strategies, and impulse control). This framed the subscales of the DERS as *abilities* or *skills*, which much better fitted the skill-related conceptualization of mindfulness in the FFMQ.

#### Meditation experience

Following a slightly adapted approach as in a previous study^32^, the practice of (1) meditation, (2) autogenic training or progressive muscle relaxation, or (3) other relaxation techniques was measured with three items (e.g., “How often do you practice meditation?”). Items were rated on 7-point scales (1 = *never*, 2 = *not regularly*, 3 = *at least once per month*, 4 = *once per week*, 5 = *twice per week*, 6 = *three times per week*, 7 = *four times per week and more*). For each participant, the highest reported value across the three items was transferred to a new variable capturing overall meditation experience (ranging from 1 = *never/not regularly*, 2 = *at least once a month,* 3 = *once per week*, 4 = *twice per week*, 5 = *three times per week*, 6 = *four times per week and more*). During this step, we combined the response options *1* = *never* and *2* = *not regularly* into one option (1 = *never/not regularly*) to ensure maximum comparability with the scale used in the previous study (associations with meditation experience on the 6-point and 7-point scales were, however, identical). Participants who reported doing some form of meditation practice were queried on the length of their meditation practice, on the time in years since they started practicing, and on the type of practice they did most in the last 6 months.

### Data analysis

The data were examined for missing values, scale score distributions, scale reliabilities, and fit of the data to the assumptions of the utilized data-analytic approach. Some score distributions were moderately skewed. Yet, due to otherwise mostly close approximations of scores to the normal distribution, the large sample size, and thus the applicability of the central limit theorem, and the similarity of results with non-parametric methods, parametric methods were used throughout for analysis. Significance was set to *p* < 0.05 (two-sided).

#### Open practices

We disclose how we determined our sample size, all data exclusions (if any), all manipulations, and all measures in the study^53^. All data, materials (variable descriptions, instructions, demographic items), and the entire analysis code are available at https://osf.io/ujp3e.

#### Structural analysis

As in previous studies^9,32,43^, the two-factor higher-order model was investigated with exploratory structural equation modeling (ESEM)^54^, fitting a two-factor model on the scale scores of the five facets of the FFMQ. ESEM is an integration and combination of the benefits of both exploratory and confirmatory factor analysis, as it allows for cross-loadings but also provides additional fit indices^54^. This approach provided insight into the factorial structure of the present data and provided sample-specific factor scores, which were used for further analysis. Analyses were conducted with the lavaan and psych packages in R^55,56^. Robust maximum-likelihood estimation (MLR) was used, as well as oblique geomin rotation. This latter method provides unbiased results, akin to confirmatory factor analysis and target-rotated solutions, without the need to specify a factor-loading pattern^57^, and, in addition, incorporates a complexity parameter which increases with the number of factors, in order to avoid inflated factor intercorrelations^54^.

The sample’s adequacy for factor analysis was checked with the Kaiser–Meyer–Olkin test and Bartlett’s sphericity test. Model fit was assessed with the comparative fit index (CFI; good fit: ≥ 0.95), the Tucker-Lewis index (TLI; good fit: ≥ 0.95), the root mean square error of approximation (RMSEA; good fit: < 0.06), and the standardized root mean squared residual (SRMR; good fit: < 0.08). Benchmarks were taken from Hu and Bentler^58^.

#### Correlation and mediation analysis

Correlation and mediation analysis was conducted with the psych and lavaan packages in R. We calculated Pearson’s* r* and Spearman’s *r*_s_ for all variable pairs. As values were very similar, only Pearson *r* values are reported. Results were interpreted according to the cutoffs proposed by Funder and Ozer^59^: *r* = 0.05 indicated a very small, *r* = 0.10 a small, *r* = 0.20 a medium, *r* = 0.30 a large, and *r* ≥ 0.40 a very large effect.

Mediation analyses examined all three DERS subscales (measuring emotional awareness, impulse control, and emotion regulation strategies) in parallel, testing for mediating effects for the associations between SRA and OTE (predictors), and cognitive and affective empathy (outcomes). In additional, mediation analysis was also performed using the five facets of mindfulness as predictors. Participant sex (using a dummy variable for this analysis step, coded 0 = female and 1 = male) and meditation experience were included as control variables. Additionally, in an exploratory analysis, multigroup structural equation modeling was used to investigate direct and indirect effects segmented by participant sex. Confidence intervals (*CI*s) of all effect estimates were obtained, using a 95% bias-corrected bootstrapping approach with 10,000 samples^60^. Statistical significance was determined using the 95% bias-corrected bootstrapping *CI*s, and standardized estimates were used to evaluate the size of the effects.

### Ethics declarations

All procedures performed in this study adhere to the ethical standards of the 1964 Helsinki Declaration and its later amendments or comparable ethical standards, and with institutional guidelines of the School of Psychology, University of Vienna. Study participation did not affect the physical or psychological integrity, the right for privacy, or other personal rights or interests of the participants. Such being the case, according to national laws (Austrian Universities Act 2002)^61^, this study was exempt from formal ethical approval.

### Informed consent

Informed consent was obtained from all individual participants included in the study.

## Results

Means, standard deviations, intercorrelations for all study variables, and measures of reliability for the scale scores, are provided in Table S1 in Supplemental Materials.

### Two-factor higher-order structure of mindfulness

Both the Kaiser–Meyer–Olkin test (KMO = 0.62) and Bartlett’s sphericity test (χ^2^[10] = 286.58, *p* < 0.001) indicated that the FFMQ data were appropriate for factor analysis. The ESEM had a good fit, χ^2^(1) = 0.38, *p* = 0.54, CFI = 1.00, TLI = 1.00, SRMR = 0.005, RMSEA = 0.000, 90% CI = [0.000, 0.087], indicating a two-factor higher-order structure similar to the one reported in previous studies^9,32,43^. Factor loadings are reported in Fig. S1 in Supplemental Materials. Observe and Describe loaded high on SRA, and Actaware and Nonjudge high on OTE. Nonreact loaded on both factors, but higher on OTE. The two higher-order factors had an intercorrelation of *r* = 0.23 which is comparable to previous studies with (mostly) nonmeditating samples^9,32^.

### Associations between mindfulness and empathy

There were two distinct patterns in the associations between the two higher-order factors of mindfulness and empathy (Table S1). SRA had a very large positive correlation with cognitive empathy (*r* = 0.44) and a small positive correlation with affective empathy (*r* = 0.11). On the subscale level, the second correlation was driven by Peripheral Responsivity and Proximal Responsivity; the association with Emotion Contagion was weaker and directionally reversed (Table S1). In contrast, OTE only had a small positive correlation with cognitive empathy (*r* = 0.09), but a medium to large negative correlation (*r* = -0.27) with affective empathy, which, on the subscale level, was driven by Emotion Contagion (larger association, compared to Peripheral Responsivity and Proximal Responsivity; Table S1).

### Mediation analysis

#### Higher-order factor level

**Figure 2:**
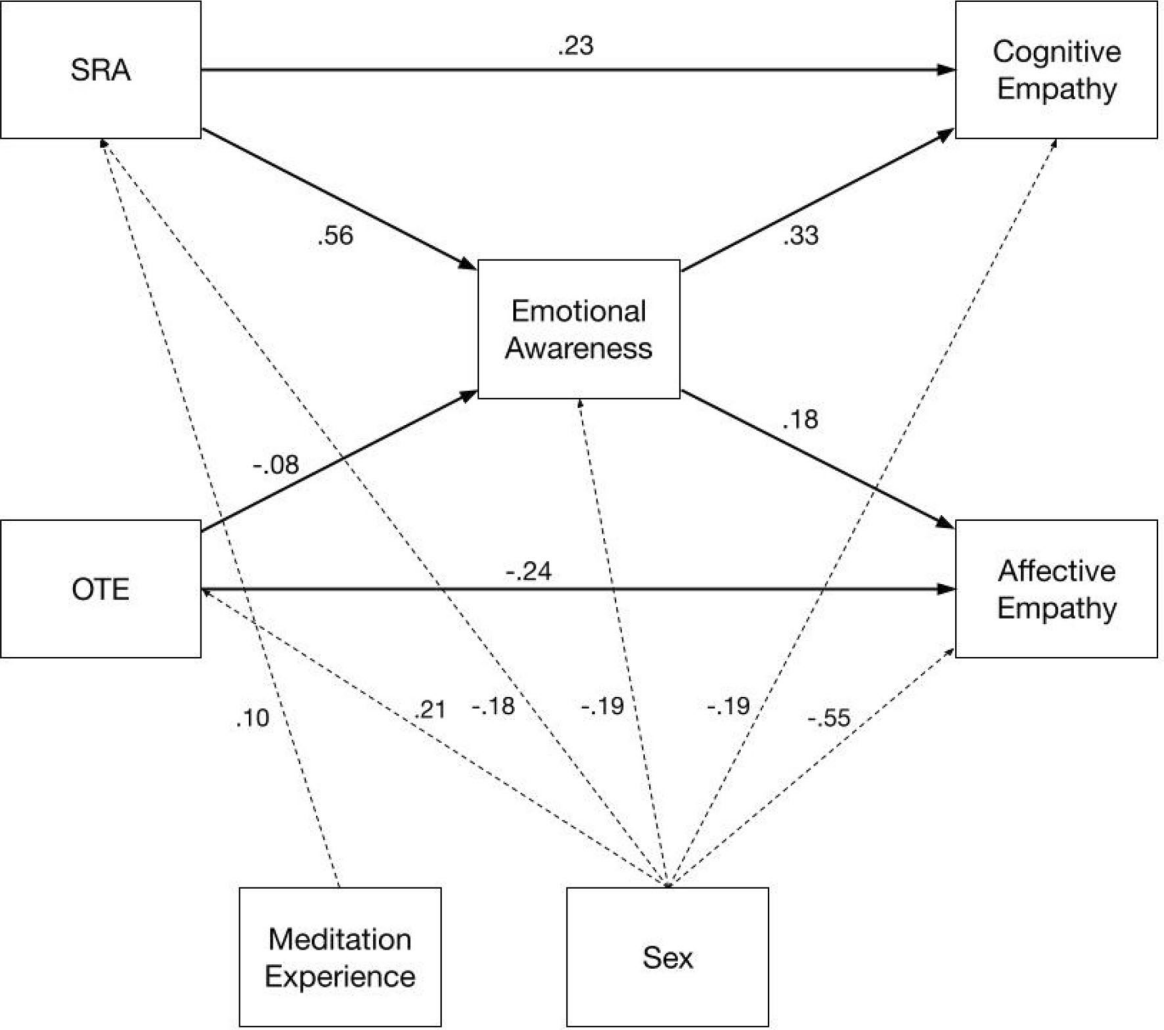
Associations between two higher-order-factors of mindfulness, emotional awareness, and cognitive and affective empathy. SRA = Self-regulated Attention, OTE = Orientation to Experience. Numbers are standardized path coefficients. Only significant direct and indirect paths (*p* < 0.05; see Table 2) and confounding effects of meditation experience and participants’ sex (see Table S3) are displayed. Significance was determined via 95% bias-corrected bootstrapping.

Controlling for participant sex and meditation experience, the total effects of SRA to cognitive and affective empathy were *r* = 0.43, 95% *CI* = [0.35, 0.52], and *r* = 0.17, 95% *CI* = [0.08, 0.25]; and of OTE to cognitive and affective empathy *r* = − 0.06, 95% *CI* = [− 0.14, 0.04], and *r* = − 0.31, 95% *CI* = [− 0.39; − 0.22]; i.e., there was no significant total effect of OTE on cognitive empathy anymore. Meditation experience had a direct effect to SRA (*r* = 0.10, 95% *CI* = [0.06, 0.15]) but no other variable, whereas participant sex had a direct effect to all variables except emotion regulation strategies (for a display of all confounder effects, see Fig. 2 and Table S3).

Mediation analysis (Table 2 and Fig. 2) indicated that emotional awareness, but neither impulse control nor emotion regulation strategies, mediated these associations significantly. Indirect effects of emotional awareness were largest for the associations of SRA with cognitive and affective empathy, but smaller, and thus evidently less relevant, for the associations of OTE with cognitive and affective empathy.

**Table 2:** Direct and indirect effects between mindfulness and empathy with aspects of emotion regulation as mediators.

Predictor → (mediator) → outcome	Estimate [95% *CI*]
Direct effects	
SRA → cognitive empathy	**0.23 [0.14, 0.33]**
SRA → affective empathy	0.08 [− 0.03, 0.18]
OTE → cognitive empathy	− 0.10 [− 0.20, 0.002]
OTE → affective empathy	**− 0.24 [− 0.35, − 0.12]**
Indirect effects	
SRA → awareness → cognitive empathy	**0.19 [0.14, 0.24]**
SRA → impulse → cognitive empathy	0.00 [− 0.003, 0.02]
SRA → strategies → cognitive empathy	0.01 [− 0.004, 0.01]
SRA → awareness → affective empathy	**0.10 [0.04, 0.16]**
SRA → impulse → affective empathy	− 0.01 [− 0.03, 0.001]
SRA → strategies → affective empathy	0.00 [− 0.02, 0.02]
OTE → awareness → cognitive empathy	**− 0.03 [− 0.05, − 0.002]**
OTE → impulse → cognitive empathy	0.03 [− 0.03, 0.08]
OTE → strategies → cognitive empathy	0.04 [− 0.03, 0.11]
OTE → awareness → affective empathy	**− 0.01 [− 0.03, − 0.002]**
OTE → impulse → affective empathy	− 0.05 [− 0.11, 0.004]
OTE → strategies → affective empathy	0.001 [− 0.08, 0.09]

SRA = Self-regulated Attention; OTE = Orientation to Experience; awareness = emotional awareness; impulse = impulse control; strategies = emotion regulation strategies. Numbers represent standardized effect estimates, alongside their bias-corrected bootstrap confidence intervals (*CI*s). Significant (*p* < 0.05) effects are printed boldface. Meditation experience and participants’ sex were included as background confounders (see Table S3).

SRA was positively associated with cognitive empathy both directly and indirectly (partial mediation), whereas with affective empathy mostly indirectly (full mediation; no significant direct effect, see Table 2 and Fig. 2). For OTE, there were tiny indirect effects, which contributed to its negative total effects on cognitive and affective empathy (Table 2).

#### Facet level

Total, direct, indirect and confounding effects obtained in the facet-level analyses are displayed in supplementary materials (Table S2; Fig. S2). There were positive total effects of Observe and Describe on cognitive and affective empathy. Actaware had a negative total effect on affective empathy and Nonreact a positive total effect on cognitive empathy, and a negative total effect on cognitive empathy. Again, only emotional awareness contributed significantly to these total effects. The largest indirect effect concerned the association of Describe with cognitive empathy (which also had the largest total effect overall), which was partially mediated via emotional awareness. The indirect effects of the associations of Observe with cognitive empathy, and of Describe with affective empathy, appeared of further relevant size. The former association was partially, and the latter association fully, mediated via emotional awareness. The remaining indirect effects were only tiny and appeared, hence, less relevant overall.

#### Multigroup analysis

In an exploratory analysis, the total, direct and indirect effects between the two higher-order factors of mindfulness and empathy were investigated segmented by sex (displayed in supplementary materials, Tables S4 and S5). Effects were similar to those across groups (see Table 2). The direct effect of SRA to cognitive empathy and its indirect effect via emotional awareness appeared to be slightly stronger in female participants, and in contrast, the direct effect of OTE to affective empathy appeared to be slightly stronger in male participants. Indirect effects between OTE and cognitive and affective empathy were tiny and statistically non-significant in analyses segmented by participant sex.

## Discussion

This study revisited—along with important extensions that went beyond prior related research evidence—the correspondence of self-reported mindfulness with empathy. We delineated two empirically-derived higher-order factors of mindfulness and, in extension to existing studies, investigated their differential associations with cognitive and affective empathy. We obtained evidence of distinct patterns of association between mindfulness and empathy, with Self-regulated Attention (SRA) being strongly related to cognitive empathy and moderately related to affective empathy. On the other hand, Orientation to Experience (OTE) had a small positive relationship with cognitive empathy, but a negative relationship with affective empathy. Further analyses on the subscale level indicated that negative associations with affective empathy mainly concerned emotional contagion. Lastly, we aimed at replicating previously reported mediational effects of emotional awareness, impulse control and emotion regulation strategies between mindfulness and empathy^26^. In the present study, only emotional awareness, but neither impulse control nor emotion regulation strategies, mediated the associations between mindfulness and empathy. Indirect effects were of a relevant size for SRA, but were less relevant for OTE.

ESEM indicated a good fit of two-higher order factors on the FFMQ data, thus replicating previous findings^32,34,43^. These results provide further evidence that the extraction of two higher-order factors leads to an efficient, empirically as well as theoretically supported, operationalization of mindfulness. Observe and Describe loaded high on SRA, and Actaware and Nonjudge on OTE. Nonreact loaded on both factors, but higher on OTE. Factor structure differences between non-meditators and meditators have been discussed in previous studies: The present study used a mixed sample (34% meditators) and was most comparable to the sample in a study of Burzler and collegues^32^. The ranking of factor loadings was largely similar in this previous study and in the present study. A single deviation occurred for Describe, which loaded only on SRA in the present study, but not on OTE, a pattern which has otherwise been reported for meditating samples^43^.

In the correlation analysis, the two higher-order factors of mindfulness showed specific associations with the two components of empathy. SRA correlated positively with cognitive empathy (Hypothesis 1) and to a lesser extent also with affective empathy, but not with emotional contagion (Hypothesis 2). Thus, SRA apparently was specifically associated with a heightened understanding of one’s own and others’ emotions in an interpersonal context, or in other words, the attentional component of mindfulness seemed to be primarily associated with cognitive aspects of empathy. It is thus possible that cognitive empathy could benefit from heightened mindful attention. Even though our cross-sectional design does not allow claims on such potential causal effects, these findings may provide inspiration for future research, and interventions specifically targeting the cultivation of SRA (as does Focused Attention meditation) could enhance interpersonal understanding.

OTE had a small positive correlation with cognitive empathy (Hypothesis 3), but a large negative correlation with affective empathy, primarily driven by emotion contagion (Hypothesis 4). High emotional contagion may be a psychological risk factor for neuroticism, alexithymia, and depression^33,36^, and associations of emotion contagion with further pathological conditions have been reported as well^35,37^. OTE thus appeared to be the main protective factor, relative to SRA, of potentially problematic aspects in the empathic process associated with emotion contagion. This finding is further backed by previous evidence of strong associations of OTE with positive mental health outcomes^43^.

These implications could also be linked to previous research on trait emotional intelligence (TEI; i.e., emotion-related dispositions and self-perceptions^31^). TEI was associated with lower emotional distress, positive mental health outcomes, and with mindfulness, respectively^29,30^. Further, mindfulness interventions seemed to promote TEI alongside subjective well-being^62^. Given the common associations, OTE may be the component of mindfulness that is specifically associated with higher TEI. In turn, TEI could also be associated with lower susceptibility to emotional contagion in the context of its role as a protective factor against emotional distress or burnout^30,63^, ultimately having a positive impact on mental health. Future research is needed to examine differential associations of the two components of mindfulness with TEI, as well as the role of emotional contagion in linking OTE to mental health outcomes, as these associations may be of clinical importance.

Interventions based on Open Monitoring meditation (which specifically benefits the cultivation of OTE) could thus protect from, or improve, symptoms in individuals with increased risk of, and susceptibility to, taking over others’ (negative) emotions. This could be of particular interest for groups frequently exposed to emotionally stressful situations (e.g., occupationally, such as counselors, social workers and health workers in general^38,64^; or in some patient populations^35^). Future studies should thus investigate the effects in such groups, as the potential benefits of mindfulness on empathy may vary among different populations. Effects could also be limited in individuals with low levels of affective empathy, such as individuals high in socially aversive personality traits^65^ (i.e., the dark triad of personality: narcissism, psychopathy, and Machiavellianism^66^). To some degree, these individuals might also be protected from psychological risks associated with a high susceptibility to emotional contagion^33^, but an extremely low level of affective empathy could completely hinder the empathic process^15^, resulting in empathic dysfunction^65^. Longitudinal studies are thus needed to clarify these relationships: Previous results of mindfulness-based interventions on trait empathy were mixed (e.g., a meta-analysis that indicated no effects in counselors^23^, and conversely reviews on effects in other healthcare professionals seemed more promising^39,67^). However, taking our cross-sectional results into account, a particular emphasis on tailor-made mindfulness-interventions and multidimensional outcome measures could result in more robust findings and potentially uncover specific pathways in interventional studies as well. Further, as changes in traits regarding emotional tendencies might manifest only after longer time periods^68^, follow-up measurements and studies applying particularly long mindfulness-programs might be needed.

In summary, the correlational findings were in line with Hypotheses 1, 2, 3, and 4 of the present study. The positive association with cognitive empathy and the negative association with emotional contagion across the two mindfulness components highlights the potential of mindfulness-based interventions to improve interpersonal understanding, without introducing a higher susceptibility to emotional contagion, thereby ultimately benefitting psychological health.

Emotional awareness was confirmed as an important mediator of the association between mindfulness and empathy. This is consistent with what has been previously reported in recent large-scale studies^24,26^. Mediational effects of emotional awareness seemed to depend on the specific higher-order factor of mindfulness: SRA (and its corresponding main subscales, i.e., Observe and Describe) were positively related with cognitive and affective empathy via emotional awareness (Hypothesis 5). Against our expectations, there were also significant indirect effects between OTE (and its corresponding main subscales, i.e., Actaware and Nonreact) and both empathy dimensions via emotional awareness, which, in contrast to SRA, were negative. However, these indirect effects for OTE were tiny and thus of small relevance. For Nonjudge, which also loaded high on OTE, no indirect effects were observed at all.

No indirect effects of impulse control or emotion regulation strategies (cf. Fuochi and Voci^26^) were observed for the associations between the two-higher order factors or the five facets of mindfulness and cognitive and affective empathy (Hypothesis 6). These null findings could stem from using a measure in the present study that better delineates empathy from related concepts and that is based on more up-to-date definitions of empathy (i.e., the QCAE) rather than the IRI, which has often been used in previous studies^26^. The IRI was based on a broad definition of empathy^11^ and may rather capture empathy-related constructs, such as emotional self-control or imagination^11,44^. This might also explain why the IRI and its scales were more strongly related to emotion regulation processes in previous studies. Furthermore, the present study controlled for additional background confounders (meditation experience and sex).

Interestingly, meditation experience only had a small direct effect on SRA, and no effect on OTE in the present study. In contrast, previous studies indicated medium effects on both SRA and OTE^32^, and increased trait mindfulness after mindfulness practice in general^69^. Yoga was by far the most common meditation technique in the present sample. Previous research indicated no large differences in trait mindfulness between different meditation techniques^70^. However, the relationship between meditation experience and trait mindfulness could depend more strongly on the duration of sessions or years of practice^71^ than session frequency. Differential associations of meditation experience and the two-higher order factors of mindfulness should be further investigated in future research.

Participants’ sex had direct effects to SRA, OTE, and cognitive and affective empathy, indicating higher scores in woman in all variables except OTE. In line with previous findings^33^, this effect was strongest for affective empathy. Associations were also investigated segmented by participant sex in an exploratory multigroup analysis, and the overall pattern of results was similar within groups compared to the results across groups.

Considering the overall weaker indirect effects explaining the associations of OTE with empathy, future studies should investigate further possible mediators in addition to those re-investigated in the current study. For example, Fuochi and Voci also reported positive indirect effects of nonattachment (a flexible way of dealing with one’s experiences and concepts of the world without clinging to them), decentering (the ability to gain a distanced perspective on one’s thoughts, to step outside one’s own perspective) and non-rumination (not showing repetitive, negative, and self-centered thoughts about the past or the future) for the associations of mindfulness facets mainly contributing to OTE and empathy^26^. Future studies on these (and other) mediators could provide additional information on differential associations of the two higher-order factors of mindfulness and empathy.

Our findings are broadly compatible with the tenets of monitoring and acceptance theory (MAT)^72^, which considers acceptance (which is comparable to OTE in the present study) a broad emotion regulation skill that is responsible for the beneficial effects of mindfulness on mental health. On the other hand, attention monitoring (which is comparable to SRA) is considered to improve selective and executive attention in affectively neutral contexts and the awareness of affective information in affective contexts (Tenet 1 and 1a of MAT); only coupled with acceptance it may also allow the efficient processing of emotional information. Otherwise, attention monitoring may exacerbate negative thoughts, feelings, or symptoms, according to MAT (Tenet 1b). The high positive association of SRA with cognitive empathy in the present study corroborates the assumption that attention monitoring improves the awareness of affective information. However, our results also suggest that there might be pathways of attention monitoring for the effective processing of emotional information specifically in empathic processes that are *independent* of acceptance and depend on the awareness of one’s own emotions only. Thus, our findings challenge Tenet 1b of MAT. This should be investigated in more detail in future studies.

In conclusion, the present study replicated findings from recent large-scale studies, but even more so provided important new insights into the differentiated relationships between the two higher-order factors of mindfulness and (sub)components of empathy. Self-Regulation Attention was strongly positively associated with cognitive empathy and Orientation to Experience negatively with affective empathy. Overall, mindfulness seemed to be associated with aspects of empathy which specifically benefit psychological health. For Self-Regulated Attention, emotional awareness could be a key mediator of these relationships. The present findings thus suggest differential roles of Self-Regulated Attention and Orientation to Experience for the links between mindfulness and empathy and highlight the importance of emotional awareness for these associations. Mindfulness interventions could be instrumental for the increase of empathy, which should be investigated in future studies based on the present results.

### Limitations

Although promising, the results of this study must be interpreted in light of some limitations. The cross-sectional data did not allow for causal conclusions. In addition, all data were based on self-reports and therefore potentially are susceptible to common-method variance and other, related biases. Longitudinal studies are therefore needed to confirm and replicate the present findings, also by beneficially considering alternative, more objective, measures (e.g., assessing empathy with behavioral tasks, as in the Multifaceted Empathy Test, which would reduce bias due to impression management and socially desirable responding^73^). Complementary measures could be of particular interest for components of affective empathy that may be partially automatic and not always dependent on conscious control^15^. For these, self-reports may provide important insights into self-perceptions^11^, but physiological measures could provide a more direct measurement of emotional reactivity^12^. Only a selection of potential mediators was examined in this study, and indirect, subscale-level effects of cognitive and affective empathy were not investigated. As well, only trait components of mindfulness and empathy were accounted for, but not their respective state components. Future inquiries along these lines might also fruitfully address the respective state components of these constructs. Meditation experience was included as a background confounder, but potential effects of meditation style, or length of meditation sessions, were not considered. Lastly, larger studies could address potential influences of mediation styles and also investigate associations on the latent level.

## Supplementary Information

Supplementary Information.

## Data Availability

The data reported in this article is available at https://osf.io/ujp3e.
