# Supplementary material for: Differential associations of the two higher-order factors of mindfulness with trait empathy and the mediating role of emotional awareness
Source: Sci Rep. 2023 Feb 24;13:3201. doi: 10.1038/s41598-023-30323-6 (PMC9958058; doi:10.1038/s41598-023-30323-6)
Supplement: Supplementary file 1 — Supplementary Information. [file 41598_2023_30323_MOESM1_ESM.pdf]

Supplementary Materials

Table S1

Means, Standard Deviations, Reliabilities, and Intercorrelations of the Factors of Mindfulness, Components of Empathy, and Aspects of Emotion Regulation

Variable	1	2	3	4	5	6	7	8	9	10	11	12	13	14	15	16	17	18	19
1. Self-Regulated Attention	—																		
2. Orientation to Experience	0.34	—																	
3. Cognitive Empathy	0.44	0.09	—																
4. Affective Empathy	0.11	-0.27	0.31	—															
5. Observe	0.61	-0.08^{ns}	0.29	0.23	—														
6. Describe	0.90	0.22	0.39	0.14	0.31	—													
7. Actaware	0.27	0.57	0.05^{ns}	-0.18	0.00^{ns}	0.16	—												
8. Nonjudge	0.24	0.94	0.04^{ns}	-0.19	-0.05^{ns}	0.13	0.36	—											
9. Nonreact	0.46	0.63	0.20	-0.26	0.09	0.24	0.26	0.40	—										
10. Perspective Taking	0.43	0.08^{ns}	0.88	0.27	0.27	0.40	0.08^{ns}	0.02^{ns}	0.17	—									
11. Online Simulation	0.33	0.08^{ns}	0.87	0.28	0.24	0.28	0.01^{ns}	0.05^{ns}	0.19	0.52	—								
12. Emotion Contagion	-0.09	-0.37	0.13	0.80	0.12	-0.04^{ns}	-0.24	-0.29	-0.35	0.10	0.13	—							
13. Proximal Responsivity	0.15	-0.18	0.37	0.85	0.24	0.15	-0.09	-0.14	-0.17	0.33	0.32	0.66	—						
14. Peripheral Responsivity	0.20	-0.06^{ns}	0.23	0.67	0.18	0.22	-0.09	-0.01^{ns}	-0.08^{ns}	0.20	0.21	0.18	0.33	—					
15. Awareness	0.56	0.11	0.48	0.24	0.36	0.53	0.01^{ns}	0.08	0.20	0.41	0.43	0.07^{ns}	0.23	0.26	—				
16. Impulse	0.25	0.57	0.10	-0.24	-0.02^{ns}	0.13	0.40	0.47	0.50	0.06^{ns}	0.13	-0.28	-0.13	-0.15	0.03^{ns}	—			
17. Strategies	0.35	0.66	0.16	-0.18	0.02^{ns}	0.23	0.45	0.55	0.56	0.14	0.13	-0.27	-0.07^{ns}	-0.07^{ns}	0.11	0.67	—		
18. Meditation experience	0.19	0.01^{ns}	0.14	0.10	0.18	0.15	-0.02^{ns}	-0.01^{ns}	0.10	0.11	0.14	0.05^{ns}	0.08^{ns}	0.09	0.18	-0.02^{ns}	0.09	—	
19. Participants' sex	-0.09	0.10	-0.16	-0.33	-0.16	-0.10	0.05^{ns}	0.05^{ns}	0.17	-0.18	-0.10	-0.25	-0.29	-0.22	-0.15	0.16	0.03	-0.04^{ns}	—
Mean	0.00	0.00	3.07	2.88	15.03	14.36	12.74	13.19	22.42	3.13	3.00	2.87	3.03	2.75	20.14	24.53	30.39	2.26	0.33
Standard deviation	0.76	0.83	0.44	0.45	3.16	3.24	3.37	3.73	4.52	0.51	0.49	0.61	0.55	0.61	5.17	4.63	6.91	1.75	0.47
Cronbach's α	—	—	0.89	0.78	0.76	0.80	0.86	0.81	0.84	0.88	0.81	0.73	0.62	0.61	0.86	0.88	0.90	—	—
McDonald's ω	—	—	0.89	0.78	0.77	0.80	0.86	0.82	0.85	0.88	0.82	0.74	0.65	0.69	0.86	0.87	0.91	—	—

Note. ^{ns} = not significant ($p \geq .05$). Subscales of the DERS (Awareness, Impulse, Strategies) were inverted for easier interpretability, participants' sex was coded 0 = female and 1 = male. Intercorrelations are Pearson correlation coefficients. Cronbach's α and McDonald's ω values are provided for all scales, except for Self-Regulated Attention and Orientation to Experience, for which factor scores were used. *SD*s and correlations of the factors obtained in ESEM were calculated from the data. All *p*s < .05, unless noted otherwise.

Table S2

Total, Direct, and Indirect Effects between Five Facets of Mindfulness and Empathy with Aspects of Emotion Regulation as Mediators

Predictor → (mediator) → outcome	Estimate [95% *CI*]
Total effects	
Observe → cognitive empathy	**0.16 [0.08, 0.25]**
Observe → affective empathy	**0.16 [0.08, 0.23]**
Describe → cognitive empathy	**0.29 [0.20, 0.38]**
Describe → affective empathy	**0.13 [0.04, 0.23]**
Actaware → cognitive empathy	-0.004 [-0.09, 0.08]
Actaware → affective empathy	**-0.12 [-0.20, -0.04]**
Nonjudge → cognitive empathy	-0.05 [-0.13, 0.04]
Nonjudge → affective empathy	-0.06 [-0.16, 0.03]
Nonreact → cognitive empathy	**0.15 [0.05, 0.24]**
Nonreact → affective empathy	**-0.23 [-0.33, 0.13]**
Direct effects	
Observe → cognitive empathy	**0.10 [0.02, 0.18]**
Observe → affective empathy	**0.13 [0.05, 0.20]**
Describe → cognitive empathy	**0.14 [0.06, 0.23]**
Describe → affective empathy	0.06 [-0.05, 0.16]
Actaware → cognitive empathy	0.01 [-0.08, 0.09]
Actaware → affective empathy	**-0.10 [-0.19, -0.003]**
Nonjudge → cognitive empathy	**-0.09 [-0.17, -0.001]**
Nonjudge → affective empathy	-0.06 [-0.17, 0.04]
Nonreact → cognitive empathy	0.08 [-0.01, 0.17]
Nonreact → affective empathy	**-0.23 [-0.33, -0.14]**
Indirect effects	
Observe → awareness → cognitive Empathy	**0.07 [0.04, 0.10]**
Observe → impulse → cognitive empathy	0.00 [-0.01, 0.004]
Observe → strategies → cognitive empathy	-0.002 [-0.01, 0.002]
Observe → awareness → affective empathy	**0.03 [0.01, 0.06]**
Observe → impulse → affective empathy	0.00 [-0.01, 0.01]
Observe → strategies → affective empathy	-0.001 [-0.01, 0.002]
Describe → awareness → cognitive empathy	**0.14 [0.10, 0.19]**
Describe → impulse → cognitive empathy	0.00 [-0.01, 0.01]
Describe → strategies → cognitive empathy	0.004 [-0.002, 0.02]
Describe → awareness → affective empathy	**0.07 [0.03, 0.12]**
Describe → impulse → affective empathy	0.00 [-0.01, 0.01]
Describe → strategies → affective empathy	0.003 [-0.01, 0.02]
Actaware → awareness → cognitive empathy	**-0.03 [-0.06, -0.01]**
Actaware → impulse → cognitive empathy	0.01 [-0.02, 0.04]
Actaware → strategies → cognitive empathy	0.01 [-0.01, 0.04]
Actaware → awareness → affective empathy	**-0.02 [-0.04. -0.004]**
Actaware → impulse → affective empathy	-0.02 [-0.05, 0.01]

Actaware → strategies → affective empathy	0.01 [-0.02, 0.04]
Nonjudge → awareness → cognitive empathy	0.01 [-0.02, 0.04]
Nonjudge → impulse → cognitive empathy	0.01 [-0.02, 0.04]
Nonjudge → strategies → cognitive empathy	0.02 [-0.02, 0.06]
Nonjudge → awareness → affective empathy	0.01 [-0.01, 0.06]
Nonjudge → impulse → affective empathy	-0.02 [-0.05, 0.01]
Nonjudge → strategies → affective empathy	0.01 [-0.03, 0.06]
Nonreact → awareness → cognitive empathy	**0.03 [0.004, 0.06]**
Nonreact → impulse → cognitive empathy	0.01 [-0.02, 0.05]
Nonreact → strategies → cognitive empathy	0.02 [-0.02, 0.06]
Nonreact → awareness → affective empathy	**0.02 [0.003, 0.04]**
Nonreact → impulse → affective empathy	-0.02 [-0.06, 0.01]
Nonreact → strategies → affective empathy	0.01 [-0.03, 0.07]

Note. Awareness = emotional awareness; impulse = impulse control; strategies = emotion regulation strategies. Numbers represent standardized effect estimates, alongside their bias-corrected bootstrap confidence intervals (*CI*s). Significant ($p < .05$) effects are printed boldface. Mediation experience and participants' sex were included as background confounders (see Table S3 for details).

Table S3

Confounding Effects of Meditation Experience and Participants' Sex in the Higher-Order and Facet-Level Mediation Analyses

Confounder → Outcome	Estimate [95% *CI*]
Higher-Order Level	
Sex → SRA	**-0.18 [-0.34, -0.01]**
Sex → OTE	**0.21 [0.04, 0.39]**
Sex → awareness	**-0.19 [-0.34, -0.04]**
Sex → impulse	**0.25 [0.10, 0.39]**
Sex → strategies	-0.03 [-0.17, 0.11]
Sex → cognitive empathy	**-0.19 [-0.35, -0.03]**
Sex → affective empathy	**-0.55 [-0.70, -0.39]**
Meditation experience → SRA	**0.10 [0.06, 0.15]**
Meditation experience → OTE	0.001 [-0.05, 0.05]
Meditation experience → awareness	0.04 [-0.002, 0.08]
Meditation experience → impulse	-0.03 [-0.07, 0.01]
Meditation experience → strategies	0.03 [-0.004, 0.06]
Meditation experience → cognitive empathy	0.02 [-0.02, 0.06]
Meditation experience → affective empathy	0.02 [-0.02, 0.06]
Facet Level	
Sex → Observe	**-0.33 [-0.50, -0.16]**
Sex → Describe	**-0.21 [-0.38, -0.04]**
Sex → Actaware	0.10 [-0.08, 0.28]
Sex → Nonjudge	0.10 [-0.08, 0.28]
Sex → Nonreact	**0.37 [0.21, 0.55]**
Sex → awareness	**-0.18 [-0.34, -0.02]**
Sex → impulse	**0.18 [0.03, 0.32]**
Sex → strategies	-0.10 [-0.24, 0.03]
Sex → cognitive empathy	**-0.21 [-0.36, -0.05]**
Sex → affective empathy	**-0.47 [-0.62, -0.32]**
Meditation experience → Observe	**0.10 [0.05, 0.14]**
Meditation experience → Describe	**0.08 [0.04, 0.12]**
Meditation experience → Actaware	-0.01 [-0.06, 0.04]
Meditation experience → Nonjudge	-0.01 [-0.06, 0.04]
Meditation experience → Nonreact	**0.05 [0.004, 0.1]**
Meditation experience → awareness	0.04 [-0.004, 0.08]
Meditation experience → impulse	-0.03 [-0.07, 0.01]
Meditation experience → strategies	0.03 [-0.01, 0.06]
Meditation experience → cognitive empathy	0.02 [-0.03, 0.06]
Meditation experience → affective empathy	0.02 [-0.02, 0.07]

Note. SRA = Self-Regulated Attention; OTE = Orientation to Experience; awareness = emotional awareness; impulse = impulse control; strategies = emotion regulation strategies. Numbers represent standardized effect estimates, alongside their bias-corrected bootstrap confidence intervals (*CI*s). Significant ($p < .05$) effects are printed boldface.

Table S4

Direct and Indirect Effects between Mindfulness and Empathy with Aspects of Emotion Regulation as Mediators in Female Participants (Group 1) in Multigroup Mediation Analysis

Predictor → (mediator) → outcome	Estimate [95% *CI*]
Total effects	
SRA → cognitive empathy	**0.46 [0.36, 0.56]**
SRA → affective empathy	**0.18 [0.07, 0.29]**
OTE → cognitive empathy	-0.04 [-0.15, 0.06]
OTE → affective empathy	**-0.30 [-0.41, -0.19]**
Direct effects	
SRA → cognitive empathy	**0.24 [0.12, 0.35]**
SRA → affective empathy	0.10 [-0.03, 0.24]
OTE → cognitive empathy	-0.07 [-0.19, 0.05]
OTE → affective empathy	**-0.24 [-0.39, -0.08]**
Indirect effects	
SRA → awareness → cognitive empathy	**0.21 [0.15, 0,29]**
SRA → impulse → cognitive empathy	0.01 [-0.01, 0.03]
SRA → strategies → cognitive empathy	0.01 [-0.01, 0.03]
SRA → awareness → affective empathy	**0.09 [0.01, 0.17]**
SRA → impulse → affective empathy	-0.01 [-0.04, 0.001]
SRA → strategies → affective empathy	0.003 [-0.02, 0.03]
OTE → awareness → cognitive empathy	-0.03 [-0.07, 0.001]
OTE → impulse → cognitive empathy	0.02 [-0.04, 0.09]
OTE → strategies → cognitive empathy	0.03 [-0.05, 0.11]
OTE → awareness → affective empathy	-0.01 [-0.04, 0.001]
OTE → impulse → affective empathy	-0.07 [-0.14, -0.002]
OTE → strategies → affective empathy	0.02 [-0.09, 0.14]
Confounding effects	
Meditation experience → SRA	**0.17 [0.06, 0.15]**
Meditation experience → OTE	0.01 [-0.05, 0.06]
Meditation experience → awareness	0.07 [-0.01, 0.08]
Meditation experience → impulse	-0.04 [-0.07, 0.02]
Meditation experience → strategies	0.06 [-0.01, 0.07]
Meditation experience → cognitive empathy	0.01 [-0.04, 0.06]
Meditation experience → affective empathy	0.02 [-0.04, 0.07]

Note. SRA = Self-regulated Attention; OTE = Orientation to Experience; awareness = emotional awareness; impulse = impulse control; strategies = emotion regulation strategies. Numbers represent standardized effect estimates, alongside their bias-corrected bootstrap confidence intervals (*CI*s). Significant (p < .05) effects are printed boldface.

Table S5

Direct and Indirect Effects between Mindfulness and Empathy with Aspects of Emotion Regulation as Mediators in Male Participants (Group 2) in Multigroup Mediation Analysis

Predictor → (mediator) → outcome	Estimate [95% *CI*]
Total effects	
SRA → cognitive empathy	**0.37 [0.21, 0.54]**
SRA → affective empathy	**0.15 [0.001, 0.31]**
OTE → cognitive empathy	-0.08 [-0.25, 0.09]
OTE → affective empathy	**-0.37 [-0.52, -0.22]**
Direct effects	
SRA → cognitive empathy	**0.21 [0.04, 0.40]**
SRA → affective empathy	0.04 [-0.12, 0.21]
OTE → cognitive empathy	-0.16 [-0.33, 0.02]
OTE → affective empathy	**-0.30 [-0.49, -0.10]**
Indirect effects	
SRA → awareness → cognitive empathy	**0.14 [0.06, 0.23]**
SRA → impulse → cognitive empathy	0.002 [-0.01, 0.03]
SRA → strategies → cognitive empathy	0.01 [-0.01, 0.07]
SRA → awareness → affective empathy	**0.12 [0.05, 0.21]**
SRA → impulse → affective empathy	-0.001 [-0.03, 0.01]
SRA → strategies → affective empathy	-0.007 [-0.06, 0.02]
OTE → awareness → cognitive empathy	-0.02 [-0.06, 0.02]
OTE → impulse → cognitive empathy	0.03 [-0.09, 0.15]
OTE → strategies → cognitive empathy	0.07 [-0.07, 0.22]
OTE → awareness → affective empathy	-0.01 [-0.06, 0.02]
OTE → impulse → affective empathy	-0.02 [-0.012, 0.10]
OTE → strategies → affective empathy	-0.03 [-0.17, 0.10]
Confounding effects	
Meditation experience → SRA	**0.21 [0.06, 0.17]**
Meditation experience → OTE	-0.01 [-0.08, 0.06]
Meditation experience → awareness	0.07 [-0.012, 0.09]
Meditation experience → impulse	-0.05 [-0.08, 0.02]
Meditation experience → strategies	0.04 [-0.02, 0.07]
Meditation experience → cognitive empathy	0.07 [-0.02, 0.09]
Meditation experience → affective empathy	0.08 [-0.01, 0.10]

Note. SRA = Self-regulated Attention; OTE = Orientation to Experience; awareness = emotional awareness; impulse = impulse control; strategies = emotion regulation strategies. Numbers represent standardized effect estimates, alongside their bias-corrected bootstrap confidence intervals (*CI*s). Significant ($p < .05$) effects are printed boldface.

Figure S1

Standardized Loadings in the Two-Factor Higher-Order Model of the FFMQ

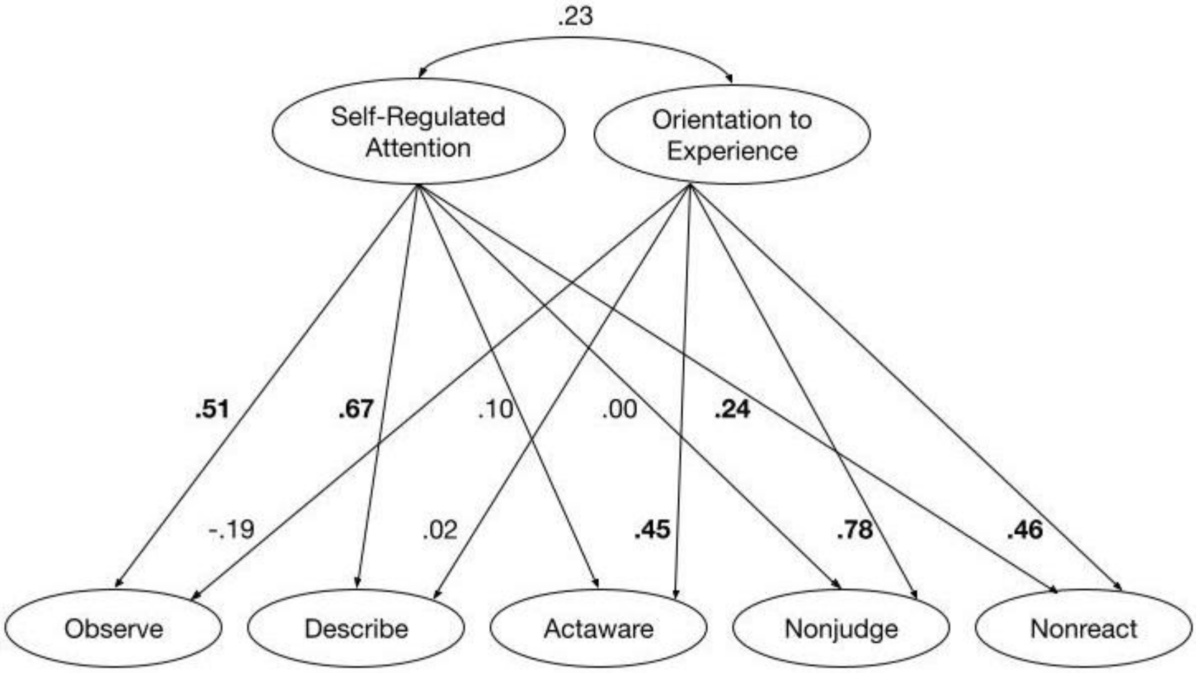

Note. Numbers represent standardized parameters. Significant ($p < .001$) parameters are printed boldface; for all other parameters, *p*s > .05.

Figure S2

Direct and Indirect Associations between Five Facets of Mindfulness, Emotional Awareness, and Cognitive and Affective Empathy

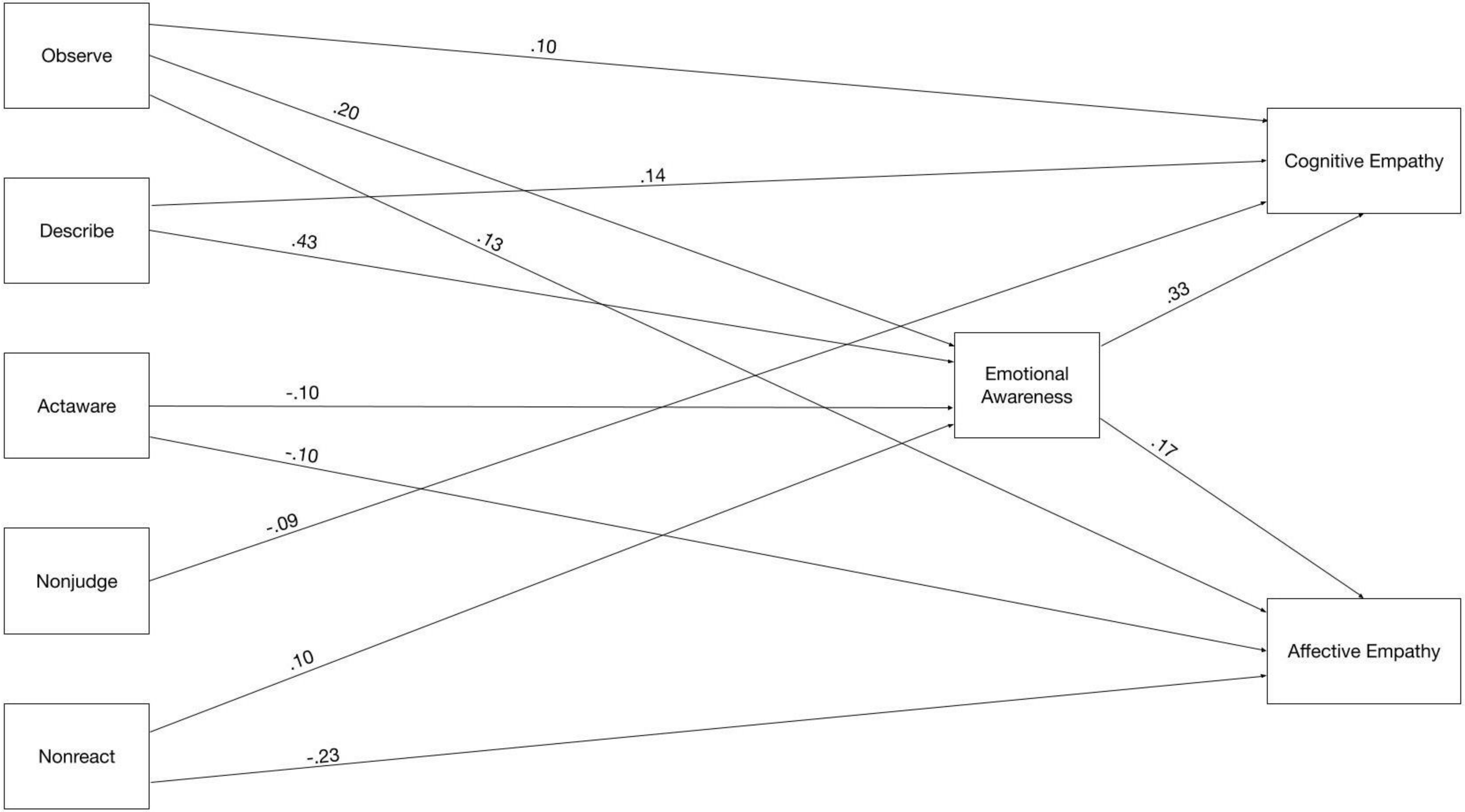

Note. Numbers are standardized path coefficients. Only significant direct and indirect paths ($p < .05$, see Table S1, and controlling for meditation experience and participants' sex, see Table S3) are displayed. Significance was determined via 95% bias-corrected bootstrapping.
